# Correction: Childhood cancer survival in the highly vulnerable population of South Texas: A cohort study

**DOI:** 10.1371/journal.pone.0303725

**Published:** 2024-05-09

**Authors:** Shenghui Wu, Yanning Liu, Melanie Williams, Christine Aguilar, Amelie G. Ramirez, Ruben Mesa, Gail E. Tomlinson

The S1–S4 Tables are uploaded incorrectly. Please view the correct S1-S4 Tables here.

## Supporting information

S1 Table South Texas Childhood Acute Lymphocytic Leukemia 5-Year Relative Survival in Different Gender and Races/Ethnicities, 1995-2017. (DOCX)

S2 Table South Texas Childhood Brain Cancer 5-Year Relative Survival in Different Gender and Races/Ethnicities, 1995-2017. (DOCX)

S3 Table South Texas Childhood Bone Cancer 5-Year Relative Survival in Different Gender and Races/Ethnicities, 1995–2017. (DOCX)

S4 Table Cancer Types by the Vital Status among South Texas Childhood Cancer Patients. (DOCX)

## References

[1] Wu S, Liu Y, Williams M, Aguilar C, Ramirez AG, Mesa R, et al. (2023) Childhood cancer survival in the highly vulnerable population of South Texas: A cohort study. PLoS ONE 18(4): e0278354. 10.1371/journal.pone.0278354 37022991 PMC10079030

