# Supplementary figures and images for: Childhood cancer survival in the highly vulnerable population of South Texas: A cohort study
Source: PLoS One. 2023 Apr 6;18(4):e0278354. doi: 10.1371/journal.pone.0278354 (PMC10079030; doi:10.1371/journal.pone.0278354)

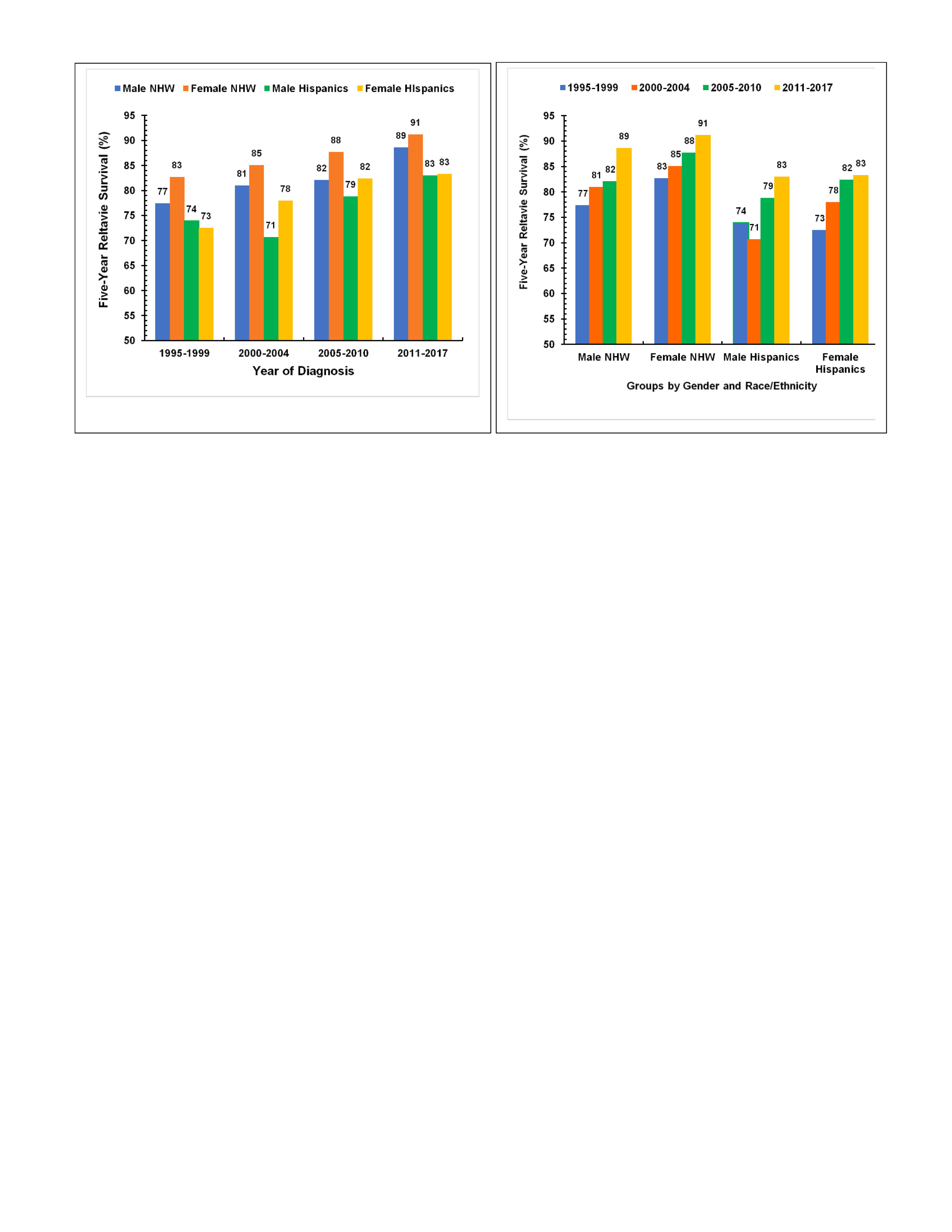

Supplement: S1 Fig — P-values for linear increasing trends of 5-year relative survival with year of diagnosis in different groups: Male NHW: P = 0.004; Female NHW: P = 0.004. Male Hispanics: P = 0.16; Female Hispanics: P = 0.09. The survival rates from female blacks showed an increasing trend from 1995 to 2017, however, the number for blacks is small and the trend was not statistically significant (P = 0.10). (TIF) [file pone.0278354.s001.tif]

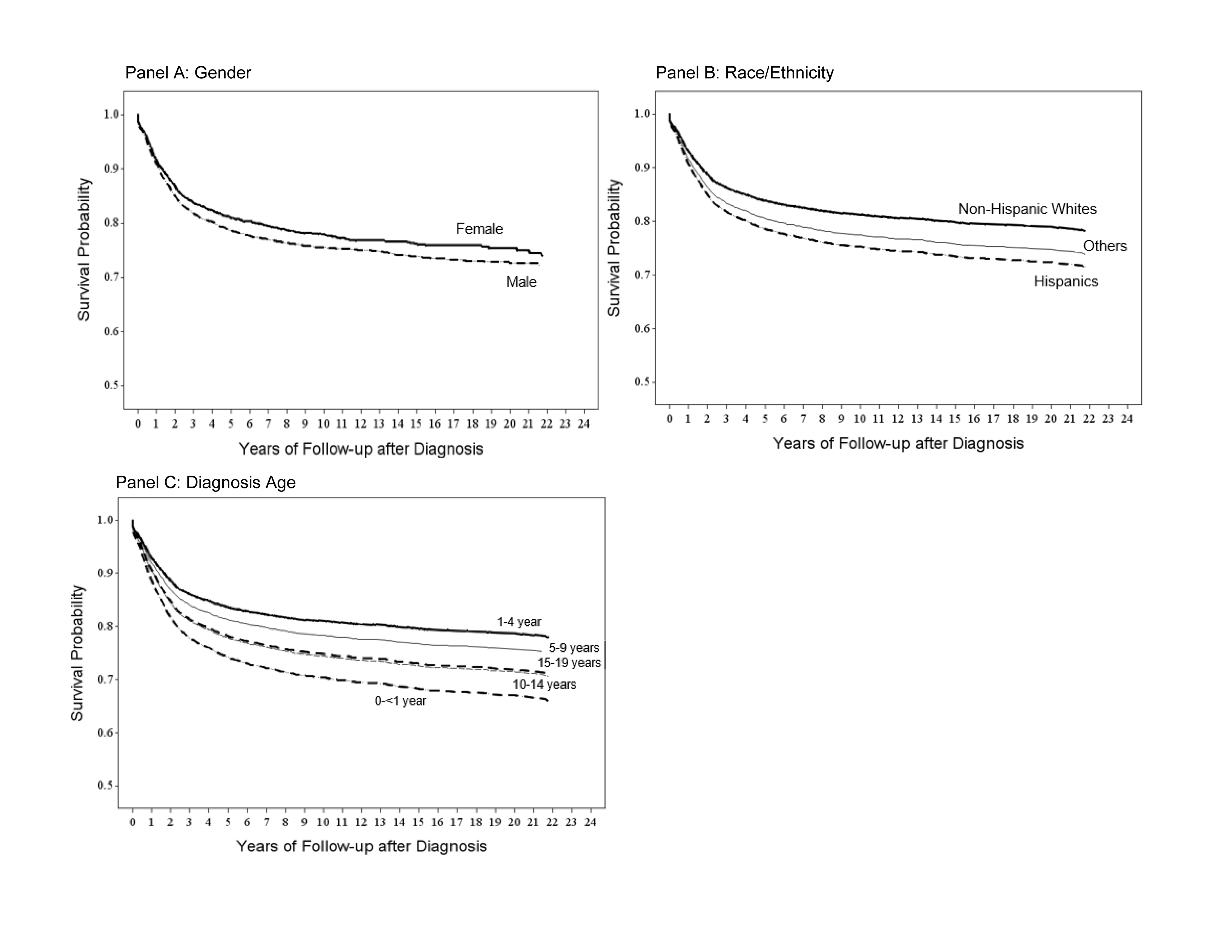

Supplement: S2 Fig — (TIF) [file pone.0278354.s002.tif]
