# Supplementary material for: Childhood cancer survival in the highly vulnerable population of South Texas: A cohort study
Source: PLoS One. 2023 Apr 6;18(4):e0278354. doi: 10.1371/journal.pone.0278354 (PMC10079030; doi:10.1371/journal.pone.0278354)
Supplement: S1 Table — (DOCX) [file pone.0278354.s003.docx]

# **S1 Table**

**S1 Table**. South Texas Childhood Acute Lymphocytic Leukemia 5-Year Relative Survival in Different Gender and Races/Ethnicities, 1995-2017

**S1 Table. South Texas Childhood** **Acute Lymphocytic Leukemia 5-Year Relative Survival in Different Gender and Races/Ethnicities, 1995-2017^a^**

| Diagnosis age and race/ethnicity | Male and female | |  | Male | |  | Female | |
| --- | --- | --- | --- | --- | --- | --- | --- | --- |
|  | N | Relative survival (SE, %) |  | N | Relative survival (SE, %) |  | N | Relative survival (SE, %) |
| 0–<1 year | |  |  |  |  |  |  |  |
| All Races | 38 | 52.4 (8.2) |  | 19 | 63.6 (11.1) |  | 19 | 40.7 (11.6) |
| NHW | 8 | 100 (0) |  | 5 | 100 (0) |  | 3 | 100 (0) |
| Hispanics | 29 | 40.5 (9.3) |  | 14 | 50.3 (13.5) |  | 15 | 30.6 (12.5) |
| Blacks | 0 | ─ |  | 0 | ─ |  | 0 | ─ |
| 1–4 years |  |  |  |  |  |  |  |  |
| All Races | 510 | 90.5 (1.3) |  | 303 | 90.1 (1.8) |  | 207 | 91 (2) |
| NHW | 96 | 90.4 (3.1) |  | 56 | 85.3 (4.8) |  | 40 | 97.5 (2.5) |
| Hispanics | 391 | 90.2 (1.5) |  | 233 | 90.7 (2) |  | 158 | 89.5 (2.5) |
| Blacks | 9 | 87.6 (11.7) |  | 5 | 100 (0) |  | 4 | 75.1 (21.7) |
| 5–9 years |  |  |  |  |  |  |  |  |
| All Races | 353 | 85.6 (2) |  | 209 | 86.9 (2.5) |  | 144 | 83.7 (3.2) |
| NHW | 57 | 88.9 (4.3) |  | 30 | 89.1 (6) |  | 27 | 88.8 (6.1) |
| Hispanics | 288 | 84.6 (2.2) |  | 174 | 86.2 (2.8) |  | 114 | 82.1 (3.7) |
| Blacks | 4 | 100 (0) |  | 3 | 100 (0) |  | 1 | 100 (0) |
| 10–14 years | |  |  |  |  |  |  |  |
| All Races | 232 | 76.9 (2.9) |  | 136 | 76.3 (3.8) |  | 96 | 77.8 (4.4) |
| NHW | 25 | 80 (8) |  | 13 | 69.2 (12.8) |  | 12 | 91.7 (8) |
| Hispanics | 199 | 75.8 (3.2) |  | 118 | 76.3 (4) |  | 81 | 75.1 (5) |
| Blacks | 3 | ─ |  | 0 | ─ |  | 3 | ─ |
| 15–19 years | |  |  |  |  |  |  |  |
| All Races | 177 | 51.1 (3.9) |  | 122 | 44.9 (4.7) |  | 55 | 64.8 (6.8) |
| NHW | 23 | 78.4 (8.6) |  | 14 | 64.4 (12.8) |  | 9 | 100 (0) |
| Hispanics | 150 | 47.7 (4.3) |  | 105 | 42.5 (5) |  | 45 | 59.9 (7.6) |
| Blacks | 3 | ─ |  | 2 | ─ |  | 1 | ─ |
| 0–19 years | |  |  |  |  |  |  |  |
| All Races | 1,409 | 77.6 (1.1) |  | 853 | 76.1 (1.5) |  | 556 | 80 (1.8) |
| NHW | 229 | 85.8 (2.4) |  | 130 | 80.4 (3.6) |  | 99 | 92.9 (2.6) |
| Hispanics | 1,136 | 75.6 (1.3) |  | 696 | 74.7 (1.7) |  | 440 | 77.1 (2.1) |
| Blacks | 19 | 75.6 (10.7) |  | 10 | 77.9 (13.9) |  | 9 | 70.1 (18.3) |

^a^ *P* values < 0.05 for the below comparisons: NHW vs. Hispanics (male and female: < 1 year, 15–19 years, and 0–19 years; male: < 1 year and 0–19 years; female: < 1 year, 1–4 years, 15–19 years, and 0–19 years); male vs. female (all races/ethnicities and NHW: 15–19 years; NHW: 1–4 years and 0–19 years). *P* values cannot be calculated for those without numbers. *P* values > 0.05 for other comparisons.  Survival rates for groups with other races were not calculated due to the small event number.
