# Supplementary material for: Childhood cancer survival in the highly vulnerable population of South Texas: A cohort study
Source: PLoS One. 2023 Apr 6;18(4):e0278354. doi: 10.1371/journal.pone.0278354 (PMC10079030; doi:10.1371/journal.pone.0278354)
Supplement: S2 Table — (DOCX) [file pone.0278354.s004.docx]

# **S2 Table**

**S2 Table.** South Texas Childhood Brain Cancer 5-Year Relative Survival in Different Gender and Races/Ethnicities, 1995-2017


**S2 Table. South Texas Childhood** **Brain Cancer 5-Year Relative Survival in Different Gender and Races/Ethnicities, 1995-2017^a^**

| Diagnosis age and race/ethnicity | Male and female | |  | Male | |  | Female | |
| --- | --- | --- | --- | --- | --- | --- | --- | --- |
|  | N | Relative survival (SE, %) |  | N | Relative survival (SE, %) |  | N | Relative survival (SE, %) |
| 0–<1 year | |  |  |  |  |  |  |  |
| All Races | 48 | 60.7 (7.1) |  | 20 | 59.7 (11.2) |  | 28 | 61 (9.3) |
| NHW | 13 | 69.7 (12.9) |  | 5 | 60.5 (22.1) |  | 8 | 75.1 (15.3) |
| Hispanics | 32 | 56.4 (8.8) |  | 12 | 57.5 (14.7) |  | 20 | 55.2 (11.2) |
| Blacks | 3 | 67.3 (27.5) |  | 3 | 67.3 (27.5) |  | 0 | ─ |
| 1–4 years |  |  |  |  |  |  |  |  |
| All Races | 200 | 69.3 (3.3) |  | 98 | 70.9 (4.7) |  | 102 | 67.7 (4.7) |
| NHW | 45 | 74.3 (6.7) |  | 22 | 86.2 (7.4) |  | 23 | 62.1 (10.8) |
| Hispanics | 149 | 66.5 (3.9) |  | 72 | 64.5 (5.7) |  | 77 | 68.4 (5.4) |
| Blacks | 4 | 100 (0) |  | 3 | 100 (0) |  | 1 | ─ |
| 5–9 years |  |  |  |  |  |  |  |  |
| All Races | 261 | 66.1 (3) |  | 138 | 66.7 (4.1) |  | 123 | 65.5 (4.3) |
| NHW | 53 | 74.9 (6.1) |  | 28 | 78.1 (8) |  | 25 | 71.5 (9.1) |
| Hispanics | 202 | 63.4 (3.4) |  | 108 | 63.3 (4.7) |  | 94 | 63.5 (5) |
| Blacks | 5 | 80 (17.9) |  | 2 | 100 (0) |  | 3 | ─ |
| 10–14 years | |  |  |  |  |  |  |  |
| All Races | 165 | 70.4 (3.7) |  | 91 | 74.8 (4.7) |  | 74 | 65.1 (5.7) |
| NHW | 44 | 76 (6.7) |  | 27 | 69.6 (9) |  | 17 | 88.3 (7.8) |
| Hispanics | 111 | 68.4 (4.5) |  | 56 | 75.6 (6) |  | 55 | 61.1 (6.7) |
| Blacks | 7 | 71.5 (17.1) |  | 5 | 100 (0) |  | 2 | 0 (0) |
| 15–19 years | |  |  |  |  |  |  |  |
| All Races | 145 | 75.4 (3.7) |  | 86 | 77.9 (4.6) |  | 59 | 71.7 (6.1) |
| NHW | 35 | 85.7 (6) |  | 21 | 85.8 (7.6) |  | 14 | 85.8 (9.4) |
| Hispanics | 102 | 71 (4.6) |  | 60 | 74.9 (5.7) |  | 42 | 65 (7.6) |
| Blacks | 7 | 85.8 (13.2) |  | 4 | 75.1 (21.7) |  | 3 | 100 (0) |
| 0–19 years | |  |  |  |  |  |  |  |
| All Races | 935 | 68.7 (1.5) |  | 499 | 70.4 (2.1) |  | 436 | 66.7 (2.3) |
| NHW | 233 | 74.6 (2.9) |  | 131 | 74.4 (3.9) |  | 102 | 74.8 (4.4) |
| Hispanics | 663 | 65.7 (1.9) |  | 340 | 67.3 (2.6) |  | 323 | 64.1 (2.7) |
| Blacks | 31 | 83.8 (6.8) |  | 22 | 91.1 (6.1) |  | 9 | 62.3 (17.8) |

^a^ *P* values < 0.05 for the below comparisons: NHW vs. Hispanics (male and female: 0–19 years; male: 1–4 years; female: 10–14 and 0–19 years). *P* values cannot be calculated for those without numbers. *P* values > 0.05 for all other comparisons. Survival rates for groups with other races were not calculated due to the small event number.
