# Supplementary material for: Childhood cancer survival in the highly vulnerable population of South Texas: A cohort study
Source: PLoS One. 2023 Apr 6;18(4):e0278354. doi: 10.1371/journal.pone.0278354 (PMC10079030; doi:10.1371/journal.pone.0278354)
Supplement: S3 Table — (DOCX) [file pone.0278354.s005.docx]

# **S3 Table**

**S3 Table**. South Texas Childhood Bone Cancer 5-Year Relative Survival in Different Gender and Races/Ethnicities, 1995–2017


**S3 Table. South Texas Childhood** **Bone Cancer 5-Year Relative Survival in Different Gender and Races/Ethnicities, 1995–2017^a^**

| Diagnosis age and race/ethnicity | Male and female | |  | Male | |  | Female | |
| --- | --- | --- | --- | --- | --- | --- | --- | --- |
|  | N | Relative survival (SE, %) |  | N | Relative survival (SE, %) |  | N | Relative survival (SE, %) |
| 0–<1 year | |  |  |  |  |  |  |  |
| All Races | 2 | 100 (0) |  | 0 | ─ |  | 2 | 100 (0) |
| NHW | 0 | ─ |  | 0 | ─ |  | 0 | ─ |
| Hispanics | 2 | 100 (0) |  | 0 | ─ |  | 2 | 100 (0) |
| Blacks | 0 | ─ |  | 0 | ─ |  | 0 | ─ |
| 1–4 years |  |  |  |  |  |  |  |  |
| All Races | 11 | 80.9 (12.3) |  | 1 | 0 (0) |  | 10 | 88.9 (10.5) |
| NHW | 1 | 100 (0) |  | 0 | ─ |  | 1 | 100 (0) |
| Hispanics | 10 | 78.8 (13.4) |  | 1 | 0 (0) |  | 9 | 87.5 (11.7) |
| Blacks | 0 | ─ |  | 0 | ─ |  | 0 | ─ |
| 5–9 years |  |  |  |  |  |  |  |  |
| All Races | 65 | 67.9 (6.2) |  | 37 | 66.7 (8.3) |  | 28 | 69.6 (9.1) |
| NHW | 12 | 60.8 (15.8) |  | 5 | 40 (21.9) |  | 7 | 85.7 (13.2) |
| Hispanics | 51 | 68 (6.9) |  | 32 | 71.9 (8.6) |  | 19 | 61.4 (11.6) |
| Blacks | 1 | 100 (0) |  | 0 | ─ |  | 1 | 100 (0) |
| 10–14 years | |  |  |  |  |  |  |  |
| All Races | 125 | 70 (4.3) |  | 68 | 74.3 (5.6) |  | 57 | 65 (6.5) |
| NHW | 22 | 79.4 (9.3) |  | 10 | 71.6 (17.1) |  | 12 | 83 (11) |
| Hispanics | 101 | 68.6 (4.8) |  | 57 | 75.9 (5.9) |  | 44 | 59.1 (7.7) |
| Blacks | 3 | 33.4 (27.2) |  | 1 | 0 (0) |  | 2 | 50 (35.4) |
| 15–19 years | |  |  |  |  |  |  |  |
| All Races | 105 | 67.6 (4.7) |  | 70 | 66.2 (5.9) |  | 35 | 70.6 (7.9) |
| NHW | 21 | 78.2 (10) |  | 15 | 68.9 (13.5) |  | 6 | 100 (0) |
| Hispanics | 78 | 63.4 (5.6) |  | 52 | 65.1 (6.7) |  | 26 | 60.2 (9.9) |
| Blacks | 4 | 75.3 (21.7) |  | 2 | 50.3 (35.6) |  | 2 | 100 (0) |
| 0–19 years | |  |  |  |  |  |  |  |
| All Races | 362 | 69.1 (2.5) |  | 213 | 67.7 (3.4) |  | 149 | 71.1 (3.8) |
| NHW | 70 | 77.4 (5.4) |  | 38 | 70.8 (8.4) |  | 32 | 84.3 (6.5) |
| Hispanics | 277 | 66.5 (2.9) |  | 166 | 67 (3.8) |  | 111 | 65.7 (4.7) |
| Blacks | 13 | 59.5 (14.2) |  | 7 | 34.6 (19.7) |  | 6 | 83.4 (15.2) |

^a^ *P* values < 0.05 for the below comparisons: NHW vs. Hispanics (female: 15–19 and 0–19 years); male vs. female (all races/ethnicities: 1–4 years; Hispanics: 1–4 years; NHW: 15–19 years). *P* values > 0.05 for all other comparisons. Survival rates for groups with other races were not calculated due to the small event number.
