# Supplementary material for: Childhood cancer survival in the highly vulnerable population of South Texas: A cohort study
Source: PLoS One. 2023 Apr 6;18(4):e0278354. doi: 10.1371/journal.pone.0278354 (PMC10079030; doi:10.1371/journal.pone.0278354)
Supplement: S4 Table — (DOCX) [file pone.0278354.s006.docx]

# **S4 Table**

**S4 Table**. Cancer Types by the Vital Status among South Texas Childhood Cancer Patients


**S4 Table.** **Cancer Types by the Vital Status among South Texas Childhood Cancer Patients**

|  | Vital status (number, percentage) | |  |
| --- | --- | --- | --- |
|  | Dead (n=1,338) | Alive (n=4,527) | Total |
| Types of cancer diagnosis |  |  |  |
| Three most common broad types based on SEER ReCode^a^ |  |  |  |
| Acute Lymphocytic Leukemia | 287 (21.45) | 1,026 (22.66) | 1,313 |
| Brain | 289 (21.60) | 533 (11.77) | 822 |
| Bone | 108 (8.07) | 200 (4.42) | 308 |
|  |  |  |  |
| Specific types based on ICCC-3 classification |  |  |  |
| I(a.1) Precursor cell leukemias | 277 (20.7) | 1,002 (22.13) | 1,279 |
| I(a.2) Mature B-cell leukemias | 11 (0.82) | 27 (0.6) | 38 |
| I(a.3) Mature T-cell and NK cell leukemias | 1 (0.07) | 3 (0.07) | 4 |
| I(a.4) Lymphoid leukemia, NOS | 1 (0.07) | 10 (0.22) | 11 |
| I(b) Acute myeloid leukemias | 104 (7.77) | 143 (3.16) | 247 |
| I(c) Chronic myeloproliferative diseases | 16 (1.2) | 124 (2.74) | 140 |
| I(d) Myelodysplastic syndrome and other myeloproliferative | 19 (1.42) | 41 (0.91) | 60 |
| I(e) Unspecified and other specified leukemias | 31 (2.32) | 73 (1.61) | 104 |
| II(a) Hodgkin lymphomas | 34 (2.54) | 287 (6.34) | 321 |
| II(b.1) Precursor cell lymphomas | 9 (0.67) | 65 (1.44) | 74 |
| II(b.2) Mature B-cell lymphomas except Burkitt lymphoma | 21 (1.57) | 85 (1.88) | 106 |
| II(b.3) Mature T-cell and NK-cell lymphomas | 14 (1.05) | 52 (1.15) | 66 |
| II(b.4) Non-Hodgkin lymphomas, NOS | 8 (0.6) | 15 (0.33) | 23 |
| II(c) Burkitt lymphoma | 9 (0.67) | 50 (1.1) | 59 |
| II(d) Miscellaneous lymphoreticular neoplasms | 11 (0.82) | 108 (2.39) | 119 |
| II(e) Unspecified lymphomas | 5 (0.37) | 31 (0.68) | 36 |
| III(a.1) Ependymomas | 36 (2.69) | 55 (1.21) | 91 |
| III(a.2) Choroid plexus tumor | 3 (0.22) | 5 (0.11) | 8 |
| III(b) Astrocytomas | 97 (7.25) | 303 (6.69) | 400 |
| III(c.1) Medulloblastomas | 46 (3.44) | 76 (1.68) | 122 |
| III(c.2) PNET | 23 (1.72) | 10 (0.22) | 33 |
| III(c.3) Medulloepithelioma | 0 (0) | 1 (0.02) | 1 |
| III(c.4) Atypical teratoid/rhabdoid tumor | 14 (1.05) | 13 (0.29) | 27 |
| III(d.1) Oligodendrogliomas | 4 (0.3) | 14 (0.31) | 18 |
| III(d.2) Mixed and unspecified gliomas | 64 (4.78) | 71 (1.57) | 135 |
| III(d.3) Neuroepithelial glial tumors of uncertain orig | 0 (0) | 4 (0.09) | 4 |
| III(e.1) Pituitary adenomas and carcinomas | 0 (0) | 2 (0.04) | 2 |
| III(e.3) Pineal parenchymal tumors | 5 (0.37) | 4 (0.09) | 9 |
| III(e.4) Neuronal and mixed neuronal-glial tumors | 1 (0.07) | 1 (0.02) | 2 |
| III(e.5) Meningiomas | 2 (0.15) | 4 (0.09) | 6 |
| III(f) Unspecified intracranial and intraspinal neoplasms | 12 (0.9) | 21 (0.46) | 33 |
| IV(a) Neuroblastoma and ganglioneuroblastoma | 55 (4.11) | 148 (3.27) | 203 |
| IV(b) Other peripheral nervous cell tumors | 0 (0) | 5 (0.11) | 5 |
| IX(a) Rhabdomyosarcomas | 59 (4.41) | 89 (1.97) | 148 |
| IX(b.1) Fibroblastic and myofibroblastic tumors | 4 (0.3) | 19 (0.42) | 23 |
| IX(b.2) Nerve sheath tumors | 16 (1.2) | 8 (0.18) | 24 |
| IX(c) Kaposi sarcoma | 1 (0.07) | 1 (0.02) | 2 |
| IX(d.1) Ewing tumor and Askin tumor of soft tissue | 7 (0.52) | 10 (0.22) | 17 |
| IX(d.10) Alveolar soft parts sarcoma | 1 (0.07) | 2 (0.04) | 3 |
| IX(d.11) Miscellaneous soft tissue sarcomas | 1 (0.07) | 7 (0.15) | 8 |
| IX(d.2) pPNET of soft tissue | 3 (0.22) | 4 (0.09) | 7 |
| IX(d.3) Extrarenal rhabdoid tumor | 2 (0.15) | 2 (0.04) | 4 |
| IX(d.4) Liposarcomas | 1 (0.07) | 9 (0.2) | 10 |
| IX(d.5) Fibrohistiocytic tumors | 1 (0.07) | 36 (0.8) | 37 |
| IX(d.6) Leiomyosarcomas | 0 (0) | 5 (0.11) | 5 |
| IX(d.7) Synovial sarcomas | 11 (0.82) | 23 (0.51) | 34 |
| IX(d.8) Blood vessel tumors | 7 (0.52) | 4 (0.09) | 11 |
| IX(d.9) Osseous & chondromatous neoplasms of soft tissue | 1 (0.07) | 2 (0.04) | 3 |
| IX(e) Unspecified soft tissue sarcomas | 17 (1.27) | 32 (0.71) | 49 |
| Not classified by ICCC or in situ | 8 (0.6) | 7 (0.15) | 15 |
| V Retinoblastoma | 6 (0.45) | 103 (2.28) | 109 |
| VI(a.1) Nephroblastoma | 12 (0.9) | 125 (2.76) | 137 |
| VI(a.2) Rhabdoid renal tumor | 1 (0.07) | 0 (0) | 1 |
| VI(a.3) Kidney sarcomas | 1 (0.07) | 6 (0.13) | 7 |
| VI(b) Renal carcinomas | 3 (0.22) | 13 (0.29) | 16 |
| VI(c) Unspecified malignant renal tumors | 0 (0) | 5 (0.11) | 5 |
| VII(a) Hepatoblastoma | 18 (1.35) | 48 (1.06) | 66 |
| VII(b) Hepatic carcinomas | 11 (0.82) | 1 (0.02) | 12 |
| VII(c) Unspecified malignant hepatic tumors | 0 (0) | 1 (0.02) | 1 |
| VIII(a) Osteosarcomas | 77 (5.75) | 104 (2.3) | 181 |
| VIII(b) Chondrosarcomas | 1 (0.07) | 12 (0.27) | 13 |
| VIII(c.1) Ewing tumor and Askin tumor of bone | 25 (1.87) | 53 (1.17) | 78 |
| VIII(c.2) pPNET of bone | 0 (0) | 2 (0.04) | 2 |
| VIII(d.1) Malignant fibrous neoplasms of bone | 0 (0) | 1 (0.02) | 1 |
| VIII(d.2) Malignant chordomas | 2 (0.15) | 6 (0.13) | 8 |
| VIII(d.3) Odontogenic malignant tumors | 2 (0.15) | 3 (0.07) | 5 |
| VIII(d.4) Miscellaneous malignant bone tumors | 0 (0) | 5 (0.11) | 5 |
| VIII(e) Unspecified malignant bone tumors | 1 (0.07) | 7 (0.15) | 8 |
| X(a.1) Intracranial & intraspinal germinomas | 7 (0.52) | 33 (0.73) | 40 |
| X(a.2) Intracranial & intraspinal teratomas | 2 (0.15) | 5 (0.11) | 7 |
| X(a.3) Intracranial & intraspinal embryonal carcinomas | 0 (0) | 1 (0.02) | 1 |
| X(a.4) Intracranial & intraspinal yolk sac tumor | 1 (0.07) | 0 (0) | 1 |
| X(a.5) Intracranial & intraspinal choriocarcinoma | 0 (0) | 1 (0.02) | 1 |
| X(a.6) Intracranial & intraspinal tumors of mixed forms | 1 (0.07) | 2 (0.04) | 3 |
| X(b.1) Germinomas: extracranial/extragonadal | 2 (0.15) | 8 (0.18) | 10 |
| X(b.2) Malignant teratomas: extracranial/extragonadal | 2 (0.15) | 11 (0.24) | 13 |
| X(b.4) Yolk sac tumor: extracranial/extragonadal | 0 (0) | 8 (0.18) | 8 |
| X(b.5) Choriocarcinomas: extracranial/extragonadal | 1 (0.07) | 11 (0.24) | 12 |
| X(b.6) Other mixed germ cell: extracranial/extragonadal | 2 (0.15) | 7 (0.15) | 9 |
| X(c.1) Malignant gonadal germinomas | 3 (0.22) | 53 (1.17) | 56 |
| X(c.2) Malignant gonadal teratomas | 4 (0.3) | 49 (1.08) | 53 |
| X(c.3) Gonadal embryonal carcinomas | 0 (0) | 30 (0.66) | 30 |
| X(c.4) Gonadal yolk sac tumor | 3 (0.22) | 29 (0.64) | 32 |
| X(c.5) Gonadal choriocarcinoma | 1 (0.07) | 4 (0.09) | 5 |
| X(c.6) Malignant gonadal tumors of mixed forms | 7 (0.52) | 120 (2.65) | 127 |
| X(d) Gonadal carcinomas | 4 (0.3) | 14 (0.31) | 18 |
| X(e) Other and unspecified malignant gonadal tumors | 5 (0.37) | 19 (0.42) | 24 |
| XI(a) Adrenocortical carcinomas | 2 (0.15) | 4 (0.09) | 6 |
| XI(b) Thyroid carcinomas | 2 (0.15) | 249 (5.5) | 251 |
| XI(c) Nasopharyngeal carcinomas | 2 (0.15) | 6 (0.13) | 8 |
| XI(d) Malignant melanomas | 8 (0.6) | 85 (1.88) | 93 |
| XI(e) Skin carcinomas | 0 (0) | 3 (0.07) | 3 |
| XI(f.1) Carcinomas of salivary glands | 2 (0.15) | 32 (0.71) | 34 |
| XI(f.10) Carcinomas of other specified sites | 6 (0.45) | 27 (0.6) | 33 |
| XI(f.11) Carcinomas of unspecified site | 5 (0.37) | 5 (0.11) | 10 |
| XI(f.2) Carcinomas of colon and rectum | 12 (0.9) | 12 (0.27) | 24 |
| XI(f.3) Carcinomas of appendix | 1 (0.07) | 26 (0.57) | 27 |
| XI(f.4) Carcinomas of lung | 4 (0.3) | 8 (0.18) | 12 |
| XI(f.5) Carcinomas of thymus | 1 (0.07) | 1 (0.02) | 2 |
| XI(f.6) Carcinomas of breast | 0 (0) | 4 (0.09) | 4 |
| XI(f.7) Carcinomas of cervix uteri | 2 (0.15) | 6 (0.13) | 8 |
| XI(f.8) Carcinomas of bladder | 0 (0) | 5 (0.11) | 5 |
| XI(f.9) Carcinomas of eye | 0 (0) | 1 (0.02) | 1 |
| XII(a.2) Pancreatoblastoma | 1 (0.07) | 0 (0) | 1 |
| XII(a.3) Pulmonary blastoma and pleuropulmonary blastoma | 1 (0.07) | 3 (0.07) | 4 |
| XII(a.4) Other complex mixed and stromal neoplasms | 1 (0.07) | 1 (0.02) | 2 |
| XII(b) Other unspecified malignant tumors | 10 (0.75) | 36 (0.8) | 46 |
|  |  |  |  |

^a^The broad cancer types were determined by the Site Recode ICD-O-3 definition [15].
